# Systematic survey of plant LTR-retrotransposons elucidates phylogenetic relationships of their polyprotein domains and provides a reference for element classification

**DOI:** 10.1186/s13100-018-0144-1

**Published:** 2019-01-03

**Authors:** Pavel Neumann, Petr Novák, Nina Hoštáková, Jiří Macas

**Affiliations:** Biology Centre of the Czech Academy of Sciences, Institute of Plant Molecular Biology, 37005 České Budějovice, Czech Republic

**Keywords:** LTR-retrotransposons, Transposable elements, Polyprotein domains, Primer binding site, RepeatExplorer

## Abstract

**Background:**

Plant LTR-retrotransposons are classified into two superfamilies, Ty1/copia and Ty3/gypsy. They are further divided into an enormous number of families which are, due to the high diversity of their nucleotide sequences, usually specific to a single or a group of closely related species. Previous attempts to group these families into broader categories reflecting their phylogenetic relationships were limited either to analyzing a narrow range of plant species or to analyzing a small numbers of elements. Furthermore, there is no reference database that allows for similarity based classification of LTR-retrotransposons.

**Results:**

We have assembled a database of retrotransposon encoded polyprotein domains sequences extracted from 5410 Ty1/copia elements and 8453 Ty3/gypsy elements sampled from 80 species representing major groups of green plants (Viridiplantae). Phylogenetic analysis of the three most conserved polyprotein domains (RT, RH and INT) led to dividing Ty1/copia and Ty3/gypsy retrotransposons into 16 and 14 lineages respectively. We also characterized various features of LTR-retrotransposon sequences including additional polyprotein domains, extra open reading frames and primer binding sites, and found that the occurrence and/or type of these features correlates with phylogenies inferred from the three protein domains.

**Conclusions:**

We have established an improved classification system applicable to LTR-retrotransposons from a wide range of plant species. This system reflects phylogenetic relationships as well as distinct sequence and structural features of the elements. A comprehensive database of retrotransposon protein domains (REXdb) that reflects this classification provides a reference for efficient and unified annotation of LTR-retrotransposons in plant genomes. Access to REXdb related tools is implemented in the RepeatExplorer web server (https://repeatexplorer-elixir.cerit-sc.cz/) or using a standalone version of REXdb that can be downloaded seaparately from RepeatExplorer web page (http://repeatexplorer.org/).

**Electronic supplementary material:**

The online version of this article (10.1186/s13100-018-0144-1) contains supplementary material, which is available to authorized users.

## Background

Long terminal repeats (LTR) retrotransposons are a very large and diverse group of transposable elements that are ubiquitous in eukaryotes. They are particularly abundant in plant genomes, making up to 75% of nuclear DNA [1]. LTR-retrotransposons replicate via an RNA intermediate (copy-and-paste mechanism), thus generating new element copies that upon integration increase the size of the host genome. There is ample evidence demonstrating that this process is among the main drivers of genome size evolution [2–5], resulting in extremely large genomes in species that are permissive to LTR-retrotransposon accumulation [6]. Although LTR-retrotransposons are often viewed as genomic parasites they may be beneficial to their hosts by providing regulatory genetic elements [7], driving rapid genomic changes [8, 9] or being an integral part of specific genome regions such as centromeres [10, 11]. Investigation of these processes is crucial to understanding genome evolution and function. Such investigations have recently become possible because of the accumulation of genome sequence data from various plant taxa. However, these efforts are complicated by the absence of a general and easily applicable system of classification for these highly diverse elements.

At present, LTR-retrotransposon are often classified only to superfamily level (the broadest category of LTR-retrotransposon classification), that includes Ty1/copia (also known as a family *Pseudoviridae* in the ICTV classification of viruses), Ty3/gypsy (*Metaviridae*), Bel-Pao (*Belpaoviridae*), retroviruses (*Retroviridae*) and endogenous retroviruses (ERV; *Retroviridae*) [12–14]. Only two of these superfamilies, Ty1/copia and Ty3/gypsy, occur in plants where they include a vast number of diverse elements. Clearly, such classifications lack detail. On the other hand, studies that divide LTR-retrotransposon sequences into families that share some minimal nucleotide sequence similarity (e.g. [12, 15]) have resulted in groups composed only of highly similar elements from closely related species. Although this approach may be useful for investigating specific species it likely misses many phylogenetic relationships between families and does not allow for comparison of retrotransposon populations from more distant taxa. In addition, due to a lack of reference databases and of clear classification guidelines, elements that should belong to the same family have occasionally been described under different names. For example, nearly identical sequences of a rice centromeric retrotransposon were designated as RIRE7, CRR1 and Osr31 [16–18]. Therefore, there is a need for a better classification system. Such a system would fill the gap between superfamily and family-based classifications by introducing an intermediate category, grouping elements across different plant taxa and better reflecting their true phylogenetic relationships.

In spite of the high diversity of their nucleotide sequences, the overall structure of LTR-retrotransposons is highly conserved. A common feature of LTR-retrotransposons is the presence of two direct repeats flanking the central region of the element (these repeats are the 5′ LTR and 3′ LTR). LTRs include sites for transcription initiation and termination and are crucial for element replication. Most LTR-retrotransposons have a primer binding site (PBS) downstream of the 5′ LTR and a polypurine tract upstream of the 3′ LTR. Upon integration LTR-retrotransposons create a target site duplication (TSD) with a characteristic length specific to each family. Intact autonomous elements encode a polyprotein that has at least five protein domains: GAG, protease (PROT), reverse transcriptase (RT), ribonuclease H (RH) and integrase (INT). Cleavage of this polyprotein by PROT domain activity releases separate mature proteins that are necessary for replication and for integration of new retroelement copies into the genome. Some of these protein sequences were found to be sufficiently conserved among all elements within Ty1/copia and Ty3/gypsy superfamilies to use them for phylogenetic analysis [19–23]. This, combined with the evaluation of specific structural features, provided the basis for phylogeny-aware classification of LTR-retrotransposons.

Most phylogenetic studies of LTR-retrotransposons performed to date have relied on the analysis of RT, RH and INT domains because they are well characterized and are relatively well conserved [19, 22, 23]. One of the most comprehensive LTR-retrotransposon phylogeny studies was carried out by Llorens et al. [20, 21] who analyzed LTR retrotransposons from a wide range of eukaryotes, including 24 Ty1/copia and 30 Ty3/gypsy elements from 26 Viridiplantae species. They identified five plant lineages of Ty1/copia elements, referred to as Oryco, Sire, Retrofit, Osser and Tork (Table 1). Ty3/gypsy elements in plants were found to belong to two major lineages, chromovirus and Tat/Athila, the former composed of the Del, Reina, CRM and Galadriel clades, the latter of the Tat and Athila clades (Table 2). Wicker and Keller [24] examined 599 Ty1/copia elements from barley, wheat, rice and *Arabiopsis thaliana*, and classified these into six ancient lineages (Maximus, Ivana, Ale, Angela, TAR and Bianca, Table 1) all of which were predicted to have existed before the divergence of monocots and dicots. However, because of the relatively small sampling of sequences from only a few species, elements assigned to the above lineages and clades represent only a small part of the LTR-retrotransposon diversity in plants. Other studies have either analyzed a population of elements in a group of closely related species [25, 26] or focused on a particular lineage of LTR-retrotransposons, e.g. chromoviruses [11, 27–31], Athila [32], Ogre [33], Tat [34] or SIRE [35–37]. Although these studies demonstrated the potential of a phylogeny-based classification their results are difficult to unify and generalize because: 1) they vary in the number of plant species included and in the number and variety of analyzed elements, 2) phylogenies were inferred from different types of data (i.e. different fragments of polyprotein sequences) and used different analysis methods, 3) they were based on limited information regarding structural and sequence features of the elements, and 4) not all studies used the same nomenclature for their elements.

**Table 1:** Unification of classification of Ty1/copia elements

REXdb	Wicker and Keller^a^	GyDB^b^	ICTV^c^
Ale	Ale	Sirevirus/Retrofit ((Koala, Melmoth, Retrofit)	pseudovirus (Melmoth, Retrofit, Hopscotch)
Alesia	Ale	–	–
Angela	Angela	–	pseudovirus (BARE-1)
Bianca	Bianca	–	–
Bryco	–	–	–
Lyco	–	–	–
Gymco-I, II, III, IV	–	–	–
Ikeros	Angela	Tork (Sto-4)	pseudovirus (Sto-4)
Ivana	Ivana	Sirevirus/Oryco (Araco, Oryco1–1, Oryco1–2, Poco, Vitico1–1)	–
Osser	–	Osser	hemivirus (Osser)
SIRE	Maximus	Sirevirus/SIRE (Endovir1–1, Opie-2, SIRE1–4, ToRTL1, TSI-9)	Sirevirus (Endovir1–1, SIRE1, ToRTL1, Opie-2)
TAR	TAR	Tork (Fourf)	–
Tork	–	Tork (Batata, RTvr2, Tnt-1, Tork4, Tto1, V12)	pseudovirus (Tnt-1, Tto1)

^a^[24], ^b^ [20, 21], ^c^ [75]

**Table 2:** Unification of classification of Ty3/gypsy elements

REXdb	GyDB	ICTV	other
chromovirus|CRM	chromoviruses|CRM (Beetle1, CRM)	–	CRM [27]
chromovirus|Chlamyvir	–	–	Chlamyvir [27]
chromovirus|Galadriel	chromoviruses|Galadriel (Galadriel, Monkey, Tntom1)	–	Galadriel [27]
chromovirus|Tcn1^a^	only Tf1/Sushi-related clades of chromoviruses in non-Viridiplantae spp.: Maggy (Maggy, Dane-1), marY1 (marY1), Pyret (Pyret, Cgret, Cft-1, Skippy), TF1–2 (TF1), V-clade (Amn-ichi, Amn-ni, Amn-san, Sushi-ichi)	–	Tcn1 [27, 31]
chromovirus|Reina	chromoviruses|Reina (Reina, Gloin, Gimli, Ifg7)	–	Reina [27]
chromovirus|Tekay	chromoviruses|Del (Del, Bagy-1, Legolas, Peabody, Retrosat-2)	Metavirus (Del1)	Tekay [27]
non-chromovirus|OTA|Athila	Athila/Tat|Athila (Athila4–1, Diaspora, Bagy-2, Cyclops-2)	Metavirus (Athila)	Athila [32]
non-chromovirus|OTA|Tat|TatI	–	–	TatI [34]
non-chromovirus|OTA|Tat|TatII	–	–	TatII [34]
non-chromovirus|OTA|Tat|TatIII	–	–	TatIII [34]
non-chromovirus|OTA|Tat|Ogre	Athila/Tat|Tat (Ogre)	–	Ogre [33]; TatIV(Ogre) and TatV [34]
non-chromovirus|OTA|Tat|Retand	Athila/Tat|Tat (Cinful-1, Tat4–1, RIRE2, RetroSor1)	Metavirus (Tat4)	Retand [65]; TatVI [34]
non-chromovirus|Phygy	–	–	–
non-chromovirus|Selgy	–	–	–

^a^Tcn1 clade belongs to Tf1/Sushi group of retrotransposons which occur in fungi and vertebrate species

We have attempted to overcome the limitations of previous studies by performing extensive searches for representative LTR-retrotransposon sequences in available green plant sequence data. These LTR-retrotransposon sequences were then classified into distinct lineages primarily based on a phylogenetic analysis of conserved domains extracted from their polyprotein sequences but also by taking into account differences in structural and sequence features of the elements. This approach eliminates problems associated with comparing highly divergent nucleotide sequences because conserved protein domains allowed us to construct meaningful alignments across all Ty1/copia and Ty3/gypsy superfamilies. The identified and classified protein domain sequences are available as a reference database in order to improve and unify future annotations of LTR-retrotransposons in plant genomes. We also compare our results to previous classification systems.

## Results

### Identification of LTR-retrotransposon

A total of 13,566 elements described in this study were predicted de novo from genomic DNA sequences of 56 Viridiplantae species using the LTR-FINDER program [38]. Predictions were based solely on structural features common to all LTR-retrotransposons, including the presence of 5′ LTR and 3′ LTR, TSDs, and 5’TG/3’CA at the element termini. Since the 5′ LTR and 3′ LTR are identical at the time of insertion of a new element copy to the genome the level of their divergence which is caused by mutations acquired over time is proportional to the insertion age. In order to retrieve sequences of relatively recently inserted elements we selected only those that had at least 95% similarity between 5′ and 3′ LTRs.

In order to be able to compare our data with sequences of previously described elements, additional LTR-retrotransposon nucleotide sequences were added from public databases [39–41] and from published studies [11, 24, 33]. In total we gathered 13,863 LTR-retrotransposon sequences, 13,795 of which were from 80 Viridiplantae species, and 68 of which originated from 36 non-Viridiplantae species including mainly fungi and metazoa. Detailed information about these sequences are provided in Additional file 1.

### Identification of conserved protein domains

In order to define protein domains suitable for phylogenetic analysis we compared the LTR-retrotransposon sequences to databases containing previously described, as well as our unpublished polyprotein domains sequences. Using a series of iterative searches we identified eight polyprotein domains, GAG, PROT, RT, RH aRH, INT and two types of chromodomains, resulting in 75,516 extracted domain sequences. The predicted domains were checked for mutual sequence similarity, similarity to sequences in the NCBI Conserved Domains Database (CDD) [42] and, if applicable, for the presence of highly conserved amino acid residues, which were reported to be important for the function [43–47] (Additional file 2). Sequences of elements that possessed protein coding domains in unexpected order, encoded multiple copies of the same type of domain or showed signs of chimerical origin were further studied and in every case removed because they were likely incorrectly predicted elements.

Based on the sequence similarity and protein domain order in putative polyprotein sequences, 5410 were classified as Ty1/copia (with the domain order GAG-PROT-INT-RT-RH) and 8453 as Ty3/gypsy (GAG-PROT-RT-RH-INT). In nearly all polyprotein sequences we identified PROT, INT, RT and RH domains (Additional files 2 and 3). However, while a GAG domain would also have been expected we could not reliably identify this domain in 442 Ty1/copia and 159 Ty3/gypsy elements. This was likely due to high levels of sequence divergence, the absence of highly conserved sites and the presence of stop codon and frameshift mutations in the coding region of many elements. In addition to the five domains above, we also identified three domains that have been reported to be specific to certain Ty3/gypsy elements. The aRH domain described by Ustyantsev et al. [34] in the Tat lineage was identified in 2941 elements. Chromodomains of the CHD and CHDCR type, which are typical of chromoviruses [11, 27, 28, 30, 48, 49], were detected in 3417 and 445 elements, respectively. Extra open reading frames (eORFs; position (in 5′ or 3′ part of the element) and orientation (forward or reverse) of the eORFs were distinguished as eORF-5’F, eORF-5’R, eORF-3’F and eORF-3’R) spanning at least 250 codons were found in 582 and 3372 Ty1/copia and Ty3/gypsy elements, respectively.

**Fig. 1:**
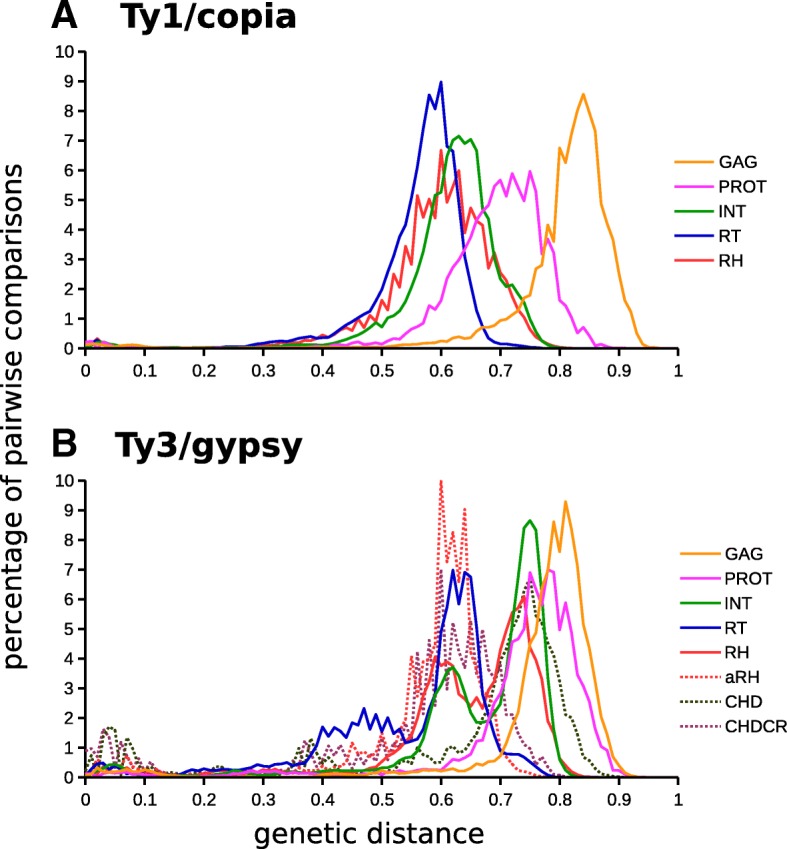
**a-b** Plots of pairwise genetic distances calculated from multiple sequence alignments of individual protein domains of Ty1/copia (**a**) and Ty3/gypsy (**b**) elements. Note that individual polyprotein domains differ considerably in their divergence and that the genetic distances of INT, RT and RH domains in Ty3/gypsy elements have bimodal distributions. The genetic distances were calculated in the program SeaView using observed distance analysis [97]

All-to-all comparisons of individual types of polyprotein domains revealed higher variability in the GAG and PROT domains compared to RT, RH and INT (Fig. 1). In addition, plots of pairwise distances for RT, RH and INT domains of Ty3/gypsy elements showed a bimodal distribution suggesting the existence of at least two markedly different groups of domains (Fig. 1b). Some polyprotein domain sequences identified in this study had either no or very weak similarity to CDD domains. This suggests that certain domain types, particularly GAG, PROT and chromodomains, are not sufficiently represented in the CDD (Additional file 2). It is also important to note that domain boundaries defined in this study differ from those in the CDD (Additional file 2). Contrary to polyprotein sequences, putative proteins encoded by the eORFs were highly heterogeneous. Based on their mutual similarity (detected using blastp and e-val < 1e-10) the eORF protein sequences found in Ty1/copia and Ty3/gypsy elements could be divided into 45 and 314 groups, respectively. Only 21.4% of the eORF protein sequences had similarity to various domain types in CDD (Additional file 4). These results suggest that the eORFs were acquired independently from multiple different sources.

### Characterization of DNA sequences of LTR-retrotransposons

The element length, TSD length and sequence, and type of PBS were identified for all predicted LTR-retrotransposons (Additional file 1). Differences in element lengths were substantial, ranging from 4.4 to 24.1 kbp. The vast majority (93.4%) of elements were flanked by 5 bp long TSDs, the remaining were flanked by 4 (6.2%) or 6 bp (0.4%) TSDs. Of the theoretical 1024 combinations of possible 5-mer sequences we found 1008, suggesting that integration of these elements to the new sites was random. Sequences of putative PBSs were highly variable, in most cases complementary to the 3′ end of various tRNAs. The most frequent were tRNA-Met (39% of all analyzed elements), tRNA-Arg (10%), tRNA-Lys (9%), tRNA-Asp (6%) and tRNA-Asn (2%). PBSs of 11% of elements were only partially complementary to tRNAs, differing from a tRNA sequence by no more to two positions. About 2% of elements possessed PBSs complementary to a half-molecule tRNA, either 1/2tRNA-Met or 1/2tRNA-Ile. Self-priming was predicted for 12% of elements and no PBS was detected in only 6% of elements. More detailed information is provided in Additional files 1 and 5.

### Phylogenetic analyses

**Fig. 2:**
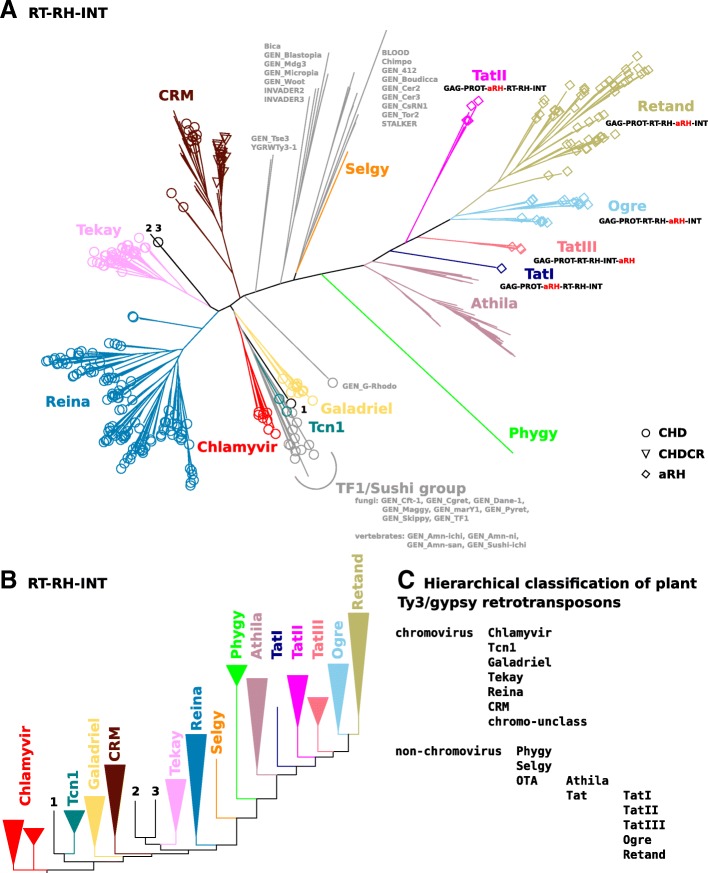
Phylogenetic trees and classification of Ty3/gypsy elements. **a** Unrooted phylogenetic tree inferred from concatenated RT-RH-INT sequences from both Viridiplantae and non-Viridiplantae elements. Branches of elements from non-Viridiplantae species are in gray. Circles, triangles and diamonds mark elements possessing CHD, CHDCR and aRH domain, respectively. Note the different position of aRH domain in the polyprotein of Tat elements. **b** Collapsed rectangular phylogram inferred from concatenated RT-RH-INT sequences from the Viridiplantae elements. Phylogenetic trees were calculated using maximum likelihood. Trees inferred from the concatenated RT-RH-INT domains were consistent with trees inferred from individual domains (Additional file 6 and data not shown). The only exception included a few chromovirus elements from *Selaginella moellendorffii* which occurred at discordant positions in the trees. Branches containing these elements are marked with numbers 1, 2, and 3 and were labeled as unclassified chromoviruses. **c** Proposed classification of Ty3/gypsy elements in plants

**Fig. 3:**
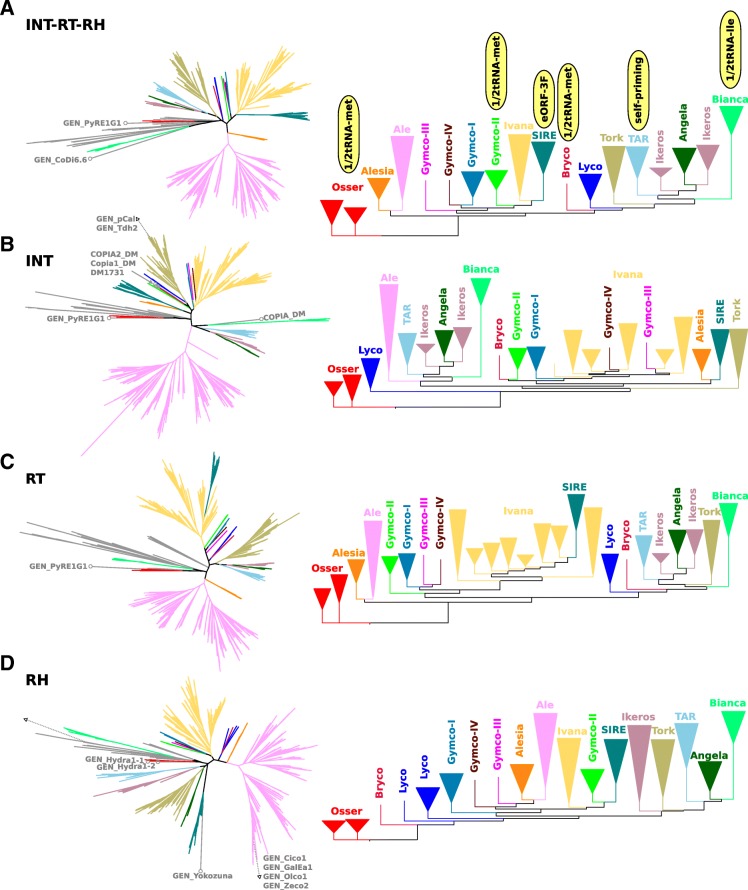
Phylogenetic trees of Ty1/copia elements. Trees were calculated using maximum-likelihood from alignments of protein sequences of concatenated INT-RT-RH (**a**), INT (**b**), RT (**c**) and RH domains (**d**). Radial phylograms on the left were inferred from datasets containing sequences of both Viridiplantae and non-Viridiplantae elements. Collapsed rectangular phylograms on the right were inferred from data sets containing only sequences from Viridiplantae species. Branches containing elements from non-Viridiplantae species are in gray. Note the discrepancies among individual trees and the relationship of some Ty1/copia groups to non-Viridiplantae elements (branches labeled with circles and names) suggesting that evolution of Ty1/copia may have involved recombination as well as horizontal transfer. All trees were rooted using the Osser clade

In order to determine evolutionary relationships among LTR-retrotransposons we inferred phylogenetic trees from alignments of RT, RH, INT and concatenated RT-RH-INT protein domain sequences. GAG and PROT sequences were excluded from the analyses because of their high heterogeneity that prevented reliable alignments. Analyses were carried out separately for Ty1/copia and Ty3/gypsy elements because protein domains from the two superfamilies differed considerably in both sequence and size and could not be reliably aligned over their entire lengths. In addition, since protein domain sequences from many elements shared high similarity, we selected a subset of representative sequences with pairwise identity over concatenated PROT-INT-RT-RH domain sequences of less than 80%. These subsets included domain sequences from 647 Ty1/copia and 358 Ty3/gypsy elements from Viridiplantae species. These sequences were supplemented with 24 Ty1/copia and 31 Ty3/gypsy elements from non-Viridiplantae species (Figs. 2 and 3, Additional file 6). Phylogenetic analyses were done using maximum-likelihood and neighbor-joining methods. Sequence similarity between some Viridiplantae and non-Viridiplantae sequences was very low, which had the potential to distort results of phylogenetic inference due to erroneous alignments, long-branch attraction, and other artifacts. Because of this, we carried out parallel analyses without the outgroup species (Figs. 2 and 3, Additional file 6).

**Fig. 4:**
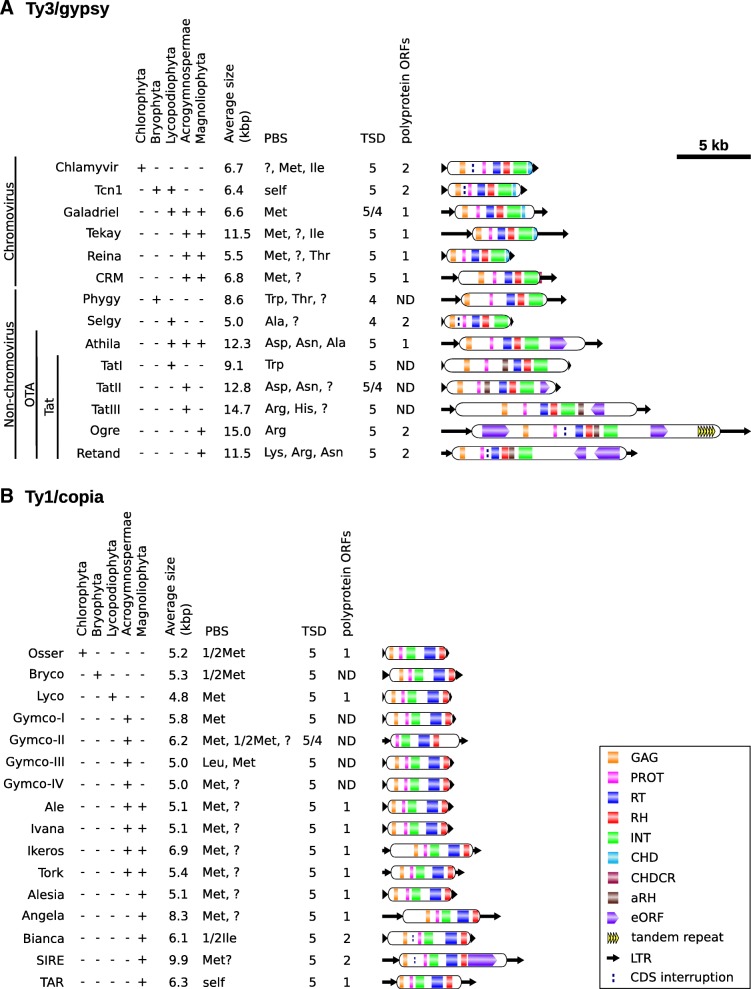
Distribution and characteristic features of individual groups of Ty3/gypsy (**a**) and Ty1/copia (**b**) retrotransposons in plants. Question mark in the “PBS type” column denotes similarity to 3′ end of undetermined types of tRNA. PBSs complementary to half-molecule tRNA are designated with a “1/2” prefix before the tRNA type. PBSs exploiting self-priming are labeled as “self”. Prevailing organization of the polyprotein coding ORFs was not determined (labeled ND) in some groups due to random stop codon and frameshift mutations in most elements. All schemes of representative elements are to scale

Individual lineages were primarily defined as groups of elements that clustered on the same branches in phylogenetic trees. In many cases lineages were also distinguished by a single or a combination of specific features that were shared by most members of a lineage. These features included the presence and position of extra protein domains (aRH, CHD or CHDCR), the presence, position and orientation of eORFs, and the type of PBS (Figs. 2 and 4 and Additional files 1, 5 and 6).

### Ty3/gypsy elements

Regardless of the type of protein domain and analysis method, Ty3/gypsy phylogenetic trees always included two major lineages we designated as chromovirus and non-chromovirus (Fig. 2, Additional file 6 and data not shown). Elements in these two lineages differed considerably from one another in the sequence of their analyzed protein domains. This explained the bimodal distribution of genetic distances shown in the Fig. 1. Besides phylogenetic distinctions, one of the biggest differences between the two lineages was the presence of the chromodomains in the vast majority chromoviruses and their absence in all non-chromoviruses (Figs. 2 and 4 and Additional files 1, 5 and 6). The majority of elements in both lineages could be further subdivided into clades which were well separated in all phylogenetic trees (Fig. 2 and Additional file 6).

Chromoviruses were classified into six clades named Chlamyvir, Tcn1, Tekay, Reina, Galadriel and CRM, these names match to groups described in previous studies [20, 27–29, 31] (Fig. 2, Table 2, Additional file 6). These clades differed considerably in some features as well as in their occurrence in various plant taxa (Figs. 2 and 4). Although CHD chromodomains were found in all clades the aromatic cage motif [50] was only detected in a significant proportion of elements of the Tcn1 (95%), Chlamyvir (59%) and Galadriel (56%) clades (Additional file 7). Plant taxonomy examination revealed that chromoviruses that have the aromatic cage motif in their chromodomain are limited to non-seed plants including algae, moss and club-moss species (Fig. 4), suggesting that the loss of this motif either preceded or occurred early in the evolution of seed plants. CHDCR chromodomains were confined to the CRM clade, but were only found in 60% of elements in this clade. The remaining members of the CRM clade either had the CHD type chromodomain (18%) or had no chromodomain (22%). Classification of chromoviruses into six clades correlates with the evolution of major taxonomic groups of plants, suggesting that chromovirus evolution in plants proceeded mainly by vertical means (Fig. 4 and Additional file 1). One possible exception to this observation was the Tcn1 clade that is composed of chromoviruses from moss and club-moss species. This clade consistently clustered on the same branch with non-plant Tf1/Sushi chromoviruses. This suggests that these chromoviruses either evolved under strong selective constrains or were transmitted by horizontal transfer (Fig. 2). Like the Tf1/Sushi chromoviruses, and unlike all other plant chromovirus clades, the majority of elements belonging to the Tcn1 clade lacked PBSs complementary to tRNAs and were predicted to exploit the self-priming mechanism of reverse transcription initiation (Additional files 1 and 5).

Non-chromovirus elements were divided into superclade OTA (composed of elements related to retrotransposons Ogre, Tat and Athila) and two species specific clades from *Physcomitrella patens* and *Selaginella moellendorffii* that were designated Phygy and Selgy respectively (Fig. 2 and Table 2; [20, 32–34, 51]). While most OTA retrotransposons were found to have eORFs and large non-coding regions, Phygy and Selgy elements were rather short and their polyprotein coding sequences spanned nearly the entire internal part (Fig. 4 and Additional files 1 and 5). The OTA superclade was split into clades Athila and Tat, the latter distinguished by the presence of an aRH domain. The position of aRH in the polyprotein varied between branches of the phylogenetic trees. The Tat clade was further divided into subclades TatI, TatII, TatIII, Ogre and Retand based on the dominant type of PBS, TSD length, and position, orientation and origin of eORF.

### Ty1/copia elements

Phylogenetic trees inferred from the alignment of INT, RT, RH and concatenated INT-RT-RH domains displayed several discrepancies in their topologies, making it difficult to reconstruct a Ty1/copia phylogeny in plants (Fig. 3). The discrepancies included Bryco, Lyco and GymcoI-IV groups representing relatively few elements from moss, club-moss and gymnosperm species, respectively, which occurred at different positions in the trees. In addition, clades Alesia and Ale clustered together, as well as Ikeros and Angela, in trees calculated from all domains but one. Ivana and SIRE elements were found on different branches in all trees except the ones inferred from the RT domain where SIRE was nested within the Ivana branch (Fig. 3). These discrepancies suggest that evolution of Ty1/copia may have involved ancient recombination events that brought together domains from elements belonging to different lineages.

In spite of the discrepancies described above, Ty1/copia retrotransposons could be divided into groups combining elements that clustered together in trees calculated from different protein domains (Fig. 3 and Table 1). Most angiosperm elements were assigned to groups that mirrored lineages defined in previous studies [20, 21, 24]. Overall, Ty1/copia elements were highly similar to each other in structure, the only features differentiating some groups included the PBS type and the presence of eORFs (Fig. 4). PBSs complementary to 1/2tRNA-Met were detected in Osser, Bryco and portion of Gymco-II elements (Additional files 1 and 5). Bianca elements had PBSs complementary to the post-transriptionally edited 1/2tRNA-Ile. The first base of this tRNA’s anticodon is changed from A to I [52], making it capable of pairing with A, U and C [53] allowing efficient binding to the PBS (Fig. 4 and Additional file 1). TAR elements were predicted to exploit self-priming and most elements from all the other groups of Ty1/copia retrotransposons had PBSs complementary to the 3′ end of the complete tRNA-Met sequence (Additional files 1 and 5). A characteristic feature of the SIRE clade was the presence of eORF-3’F which was detected in 74% of elements of this group. eORF-3’F sequences showed high heterogeneity on both DNA and protein sequence levels, suggesting that they either have different origins or have evolved very fast. In order to remove any doubts regarding the classification of SIRE elements, we compared the protein domains of all Ty1/copia elements included in this study with sequences downloaded from MASiVEdb, the most comprehensive database of these elements [36]. From a total of 17,594 elements downloaded from this database, 17,588 (99.97%) had their best hit to protein domains from elements classified in this study as SIRE, confirming that our classification of SIRE is in agreement with that of Bousious et al. [36].

### REXdb database

All 75,516 polyprotein domain sequences identified in this study were associated with element classification information and used to create a database primarily designed for use within the RepeatExplorer pipeline [54, 55], hence the name RepeatExplorer database (REXdb). The database consists of protein sequences in FASTA format and a table providing classification of entire elements. It can be downloaded from the RepeatExplorer web page [56] and used for similarity searches separately from the pipeline. Individual types of polyprotein domain sequences in the database are distinguished by a prefix followed by the element name.

## Discussion

LTR-retrotransposons are likely to have been present in Viridiplantae genomes since their origin approximately 700–1500 million years ago [21, 57]. Such a long period of evolution has produced a huge number of variants, most of which share no or very little similarity on a DNA sequence level. On the other hand, polyprotein sequences evolution was likely constrained by the necessity to retain function. Consequently, some polyprotein domains share significant sequence similarity even among elements that are separated by hundreds of millions years, making them ideal for phylogenetic studies. Most phylogenetic studies of LTR-retrotransposons have relied on the analysis of RT, RH and INT domains because they are well characterized and relatively well conserved [19, 22, 23]. Although previous studies on the phylogeny of LTR-retrotransposons in plants demonstrated that they diverged into a few phylogenetically distinct groups [11, 20, 21, 24, 27, 29–35, 51], it had remained unclear whether phylogenies inferred from different polyprotein domains were congruent and suitable for better a classification of complete elements. This study represents the first attempt to classify LTR-retrotransposons from a wide range of plant species using phylogenetic analysis of multiple protein domains. We correlated our results with various sequence and structural features, including the presence and position of extra domains in the polyprotein, the presence, position and orientation of eORFs, and the type of PBS.

The hierarchical classification of Ty3/gypsy retrotransposons proposed in this study was supported not only by the phylogenies inferred from the analysis of three polyprotein domains but also by a combination of features characteristic of individual clades. These results are consistent with partial phylogenies in previously published studies (Table 2). On the other hand, discrepancies between phylogenetic trees generated for different domains of Ty1/copia retrotransposons hampered their classification into hierarchically ordered groups. Similar discrepancies, but using a much smaller dataset, were obtained by Llorens et al. [21]. Therefore, in their study they assigned taxonomic levels based on phylogenies from concatenated PROT-INT-RT-RH sequences. Although our trees inferred from concatenated alignments of INT-RT-RH domains were consistent with Llorens et al. [21] and with the tree published by Wicker and Keller [24] based on approximately 500 amino acids covering the RT domain, discrepancies between phylogenies inferred from individual domains observed in our study suggests that some previously defined lineages of Ty1/copia retrotransposons may have a chimeric origin due to ancient recombination events between ancestral elements. Therefore, we classified Ty1/copia retrotransposons into groups based on phylogenetic tree clustering that were consistent among trees based on different domains (Table 1).

### Significance of sequence and structural features

Although LTR-retrotransposons were primarily classified based on the phylogeny of RT, RH and INT domains, certain sequence and structural features were highly characteristic of some groups of elements. These features are not only classification criteria but also reflect important biological distinctions between individual groups of elements.

Chromodomains were found in 94% of chromoviruses but none were found in any other group of LTR-retrotransposons included in this study, suggesting that the presence of this domain can be considered a highly reliable classification feature. Chromodomains are assumed to provide chromoviruses with targeting preferences [11, 27–30, 48, 49] and have previously been classified into types I, II, and CR motif [49]. Types I and II have sequence and structural similarity both to each other and to cellular chromodomains, such as those present in HP1 or Swi6 proteins [49]. While type I and cellular chromodomains possess three sites of highly conserved amino acid residues (Phe, Tyr or Trp) forming an aromatic cage that binds a methylated lysine on histone H3 (H3K9) [49, 50], the type II chromodomains lack these residues at the first and usually also the last of these sites and their interacting partner(s) has not yet been identified [30, 49]. In this study we treated type I and II chromodomains as a single group because previous classification efforts did not sufficiently reflect their sequence divergences and potential differences in function. However, our results suggest that the aromatic cage motif is preserved in elements from non-seed plant species (including algae, mosses and club-mosses) but absent in chromoviruses from seed plants. It is conceivable that the evolution of chromodomains has led to the adaptation of chromoviruses to changing chromatin environment in plants or may have contributed to functional diversification of clade specific chromodomains as proposed by Novikov et al. [29]. The highest level of adaptation to a particular chromatin type was attained by chromoviruses possessing the CR motif chromodomain that are preferentially targeted to centromeres [11]. This type of chromodomain, in this study designated as CHDCR, is specific to the CRM clade of chromoviruses and has no sequence similarity to type I and II chromodomains. Although CRM elements occur in both gymnosperm and angiosperm species those with CHDCR chromodomain have so far been identified only in the latter group of species (Additional file 1 and [11, 27, 28, 48]), suggesting that they are evolutionarily younger. Because of the lack of similarity between CHD and CHDCR chromodomains and the different positions of their coding sequences (Fig. 4) we concluded that the CHDCR chromodomain is more likely to have been acquired from an unknown source rather than having evolved from a CHD domain and that the event was linked to the loss of the CHD chromodomain.

The presence of two RH domains is a unique feature of the Tat lineage. Like all other LTR-retrotransposons Tat elements possess an RH domain following the RT domain but they also have an additional RH domain, referred to as aRH, which occur in the polyprotein at three different positions (Figs. 2 and 4 and [34]). The aRH domain has been suggested to have a polyphyletic origin, having been acquired independently at least three times in the evolutionary history of Tat LTR-retrotransposons, followed by the degeneration of the catalytic core of the original RH domain [34]. The position of the aRH domain fully correlates with further sub-classification of the Tat lineage into five clades and with the known species distribution. The presence of the aRH domain between PROT and RT domains is characteristic of TatI and TatII clades which are composed of elements from club-mosses and gymnosperms species respectively. The gymnosperm species also carry TatIII elements possessing an aRH domain after the INT domain. On the other hand, Ogre and Retand elements, found in angiosperm species, have an aRH domain between their RH and the INT domains. It should be noted that our classification of Tat elements into the five subclades differs from the study of Ustyantsev et al. [34] where six subclades were distinguished (Table 2).

Large proportions of elements in some lineages of LTR-retrotransposons were found to possess eORFs, however, unlike the polyprotein, putative protein sequences of these eORFs were highly divergent and possessed no conserved domains common to most elements of the same lineage. For example, although 74% of SIRE elements possessed eORF-3’F, the putative protein sequences could be divided into 19 groups that share no or very little mutual similarity (blastp e-val > 1e-10). This suggests that these eORFs either evolved very fast or have been acquired independently from different sources, raising the question whether they have any function. Although the applicability of protein sequences encoded by the eORFs for classification of LTR-retrotransposons is limited, some of them do posses domains that are specific for a significant proportion of elements of given lineage. The most prominent of these are the plant mobile domain (pfam10536), Transposase_28 (pfam04195) and Athila ORF-1 (pfam03078) that were found in 57, 23 and 16% of Ogre, Retand and Athila elements respectively (Additional file 4).

The shortest, yet very important, feature of LTR-retrotransposons is a PBS sequence located adjacent to the 3′ end of the 5′ LTR, that is complementary to 3′ end of primer molecule initiating reverse transcription. In this study we found that plant LTR-retrotransposons exploit all three types of primers described previously [58] and that the type of the primer is in most cases characteristic of individual lineages (Fig. 4 and Additional files 1 and 5). The primer is either: 1) a complete molecule of a mature tRNA, 2) a half-molecule tRNA generated by cleaving the tRNA in the anticodon stem or, 3) a self primer released by cleavage of the 5′ LTR of the retrotransposon transcript. The majority of lineages of both Ty1/copia and Ty3/gypsy elements exploit mature, complete tRNAs but they differ in the tRNA variant. The most frequent variant is tRNA-Met, which was found in all Ty1/copia lineages exploiting complete tRNAs as a primer and in most chromoviruses. On the other hand, tRNA-Met is not used by non-chromoviruses which exploit a number of different tRNAs, mainly tRNA-Ala, tRNA-Arg, tRNA-Asp, tRNA-Lys and tRNA-Trp. The half molecule tRNAs were found to be exploited exclusively by Ty1/copia elements belonging to Osser, Bryco, Gymco-II (all using 1/2tRNA-Met) and Bianca (1/2tRNA-Ile) lineages. The use of self-primers have previously been described in Tf1/Sushi group of Ty3/gypsy LTR-retrotransposons from yeast and vertebrates [59–61] and have also been proposed for Houba and Osr-1 Ty1/copia families in plants [62]. Consistent with these studies we found that self-priming is likely exploited by elements belonging to the Tcn1 and TAR lineages, the former being phylogenetically related to the Tf1/Sushi group (Fig. 2) and the latter including, among others, Houba and Osr-1.

Other features examined in this study, including the element size, TSD length and sequence, and the structure of the polyprotein coding region appeared to be relatively insignificant for classification due to the high level of intralineage or low level of interlineage variability. In general, the shortest elements were found in lineages lacking eORFs which ranged in average size between 4.8 and 11.5 kb and had their internal region almost entirely composed of the polyprotein coding sequence (Fig. 4 and Additional files 1 and 5). On the other hand, the largest elements, reaching sizes up to about 24 kbp, belonged to non-chromoviruses. In many cases these elements possessed not only eORFs but also extremely large LTRs (each up to about 5 kb) and expanded non-coding regions which often included arrays of tandem repeats (Fig. 4 and [63–66]). The length of TSDs was found to be characteristic of most lineages, either 4 or 5 bp. With the exception of the Phygy elements that have an insertion preference to NCGN sequence motif, the TSD sequences were highly variable suggesting that most LTR-retrotransposons in plants do not recognize particular sequences during integration. Although the organization of the polyprotein coding region into one or more ORFs (either immediately consecutive or overlapping) has been reported to be important for the regulation of expression of individual proteins of the polyprotein [67], the random occurrence of stop codons and frame-shift mutations in most elements suggested that they were non-functional. Nevertheless, analysis of elements with intact coding sequence revealed that most groups of LTR-retrotransposons have one type of domain organization that largely prevails over the others (Additional file 5). While most groups encoded their entire polyprotein into a single ORF some groups possessed two ORFs. The first ORF encoding GAG or GAG-PROT domains, while the second ORF encoded all the remaining domains. Interestingly, Bianca and Ogre elements were found to have two ORFs that were separated by, on average, 172 and 274 bp long regions respectively. This suggests that they evolved a unique strategy for the regulation of polyprotein expression. Previous experimental work has shown that in Ogre elements from *Pisum sativum* and *Medicago truncatula* the region between GAG-PROT and RT-RH-INT ORFs is an intron that is spliced out from only a subset of transcripts in order to allow translation of the entire polyprotein coding sequence [68, 69].

### A unified classification and nomenclature to prevent confusion

The large scale of this study and the inclusion of previously described elements allowed us to unify previously used classifications and names of individual groups of elements, as well as to reveal discrepancies in classification and nomenclature among this and the other studies (Tables 1 and 2). SIRE element names were the most confusing. We named the SIRE lineage after the first described element [70] and according to the classification of Llorens et al. (2009, 2011) [20, 21]. However, elements belonging to this lineage have also been described as endogenous retroviruses [71, 72], Sireviruses [35, 36, 73], Agroviruses [74] or as belonging to the Maximus lineage [24]. In the GyDB database [20, 21] the term Sirevirus was misleadingly reserved for a group of elements belonging not only to the SIRE lineage but also to the Oryco and Retrofit lineages which correspond, respectively, to the Ivana and Ale lineages described here as well as in Wicker and Keller [24]. The term “endogenous retrovirus” [32, 51, 71, 72] has been used for two distinct groups of plant LTR-retrotransposons, (designated here as SIRE and OTA) neither of which is related to genuine endogenous retroviruses in vertebrate genomes. It has been speculated that eORFs, located downstream of the polyprotein-coding region in many SIRE and OTA elements, may have functions analogous to the retrovirus env gene. However, since retroviruses have never been detected in plants and the function of the eORFs is rather speculative we propose that the designation of any group of plant LTR-retrotransposons as retroviruses should be avoided. Comparison of our classification with the ICTV taxonomy [75] revealed that the three genera of Ty1/copia elements (Pseudoviridae) do not reflect phylogenetic relationships and that all Ty3/gypsy (Metaviridae) elements from plant species belong to the genus Metavirus (Tables 1 and 2). Thus, the current version of the ICTV classification at the genus level is obsolete and not suitable for plant LTR-retrotransposons.

### Species distribution of individual groups of LTR-retrotransposons in plants

Species distributions differed considerably among individual groups of LTR-retrotransposons. In agreement with the previous studies suggesting that chromoviruses represent the oldest and the most widespread lineage of Ty3/gypsy retrotransposons [21, 27] we found these elements in all major groups of species analyzed in this study. However, individual clades of chromoviruses had limited distributions. While Galadriel elements were widely distributed in various Tracheophyta species, Tekay, Reina and CRM elements were found only in Spermatophyta, and Chlamyvir was specific to algae (Fig. 4). Strikingly, plant elements belonging to the Tcn1 clade, which occurred only in moss and club-moss species, appeared to be closely related to the Tf1/Sushi group of LTR-retrotransposons that is composed of various families from fungi and vertebrates [59, 76, 77]. Previous studies found that RT-INT fragment sequences of plant Tcn1 representatives share unexpectedly high similarity with Tcn1 retrotransposon from the fungus *Cryptococcus neoformans* [77], suggesting that elements of this clade either evolved under strong selective constrains or were transmitted by horizontal transfer [29, 31]. We found that other polyprotein domains share much lower similarity (data not shown), indicating that high similarity between the RT-INT sequences is at least partially due to stronger selective constrains acting on these domains. On the other hand, the hypothesis of ancient horizontal transfer of Tf1/Sushi elements is strongly supported by their limited occurrence in plants and by the self-priming mechanism of reverse transcription initiation which is likely to be common for Tf1/Sushi elements but is not exploited by any other group of plant chromoviruses (Additional files 1 and 5 and [59–61]). In contrast to chromoviruses, non-chromoviruses were not found in algae, suggesting that they either evolved later in the evolution of plants or were lost in algae. Although chromodomain-lacking lineages of Ty3/gypsy retrotransposons also exist in non-plant species [20, 21] their relationship to plant non-chromoviruses remains unclear. Individual lineages of Ty1/copia had narrower distribution among plant taxa than Ty3/gypsy (Fig. 4), which can at least partially be due to their complicated pattern of evolution that prevented hierarchical classification.

## Conclusions

In this study we showed that, despite their enormous DNA diversity, plant LTR-retrotransposons can be reliably classified using phylogenetic approaches into a small number of groups. Our proposed classification relies on phylogenetic analysis of the RT, RH, and INT domain sequences but in many cases is strongly supported by structural and sequence features. These include the presence and position of extra domains in the polyprotein, presence, position and orientation of eORFs, and the types of PBS. These features reflect biologically important distinctions among individual groups of elements and emphasize the need for the more detailed classification. Our database of protein domain sequences from classified elements is the most comprehensive dataset of its kind, representing a suitable reference for a unified classification of LTR-retrotransposons in plants. Since the database has a simple structure and is open for use and improvements by the scientific community we expect it to be continuously developed as new sequence data, especially from under-represented taxa of non-seed plants, becomes available.

## Methods

### Identification of LTR-retrotransposons

Genomic DNA sequence data were downloaded from Phytozome [78] and Dendrome [79] databases (Additional file 1). LTR-retrotransposon sequences were predicted using LTR-FINDER program [38]. LTR length was set to 100–6000 bp, distance between 5′ and 3′ LTRs was set to 1000–20,000 bp, minimum similarity between 5′ and 3′ LTRs was set to 95% and only sequences that were flanked by TSDs and had TG and CA at 5′ and 3′ ends of LTRs, respectively, were scored (LTR-FINDER parameters -l 100 -L 6000 -d 1000 -D 20000 -S 5.00 -F 11111000000 -M 0.95 -w 2). The set of predicted elements was further filtered to remove sequences that had more than ten Ns, contained nested insertion(s), lacked the polyprotein coding region or were redundant.

### Identification of protein domains

Protein domains were identified using iterative searches for similarity to our in-house database of protein domains and to protein domain sequences deposited in conserved domain database (CDD; [80, 81]. Searches were carried out separately for Ty1/copia and Ty3/gypsy elements and included both protein-protein and DNA-protein comparisons using the appropriate blast (blastp, blastx), fasta (fasta36 and fasty36) and last programs [82–86]. After each iteration, predicted protein domains were aligned using muscle [87] and alignments were inspected and manually edited in SeaView [88]. Verified protein domains sequences were used as a database for the next iteration and this was repeated until no more sequences were identified. The set of all polyprotein domains identified in this study is provided in Additional file 3 and can be downloaded from the RepeatExplorer web page [56] or as a part of RepeatExplorer software package [89].

### Other bioinformatic analyses

Computer analyses were performed using custom BioPerl [90] and R [91] scripts or the external programs specified next. tRNA sequences were predicted using tRNAscan-SE [92] in all genomic sequences that were used for identification of LTR-retrotransposons. A few additional sequences of plant tRNAs were added from the genomic tRNA database [93]. Since all mature tRNAs have CCA at their 3′ end, which was missing in the sequences predicted in the genomic sequences because it is added post-transcriptionally, it was added to every sequence manually. Identification of putative PBSs was performed using blastn searches followed by selecting for only perfect matches from 50 bp regions downstream of 5’ LTR to at least 12 bp from 3′ ends of tRNAs. Sequences that lacked a perfect match to the 3′ end of tRNA but possessed TGG 0–5 bp downstream of the 3′ end of 5′ LTR were tested to see whether sequences starting with their particular tri-nucleotide were similar to the 3′ end of a tRNA. This was done using PatMaN program [94], allowing for up to two differences between the query and the hit, one of which could be an indel. Putative self-primer sequences in the 5′ LTR were detected using blastn as regions complementary to at least 10 bp long region starting 0–5 bp downstream of the 3′ end of 5′ LTR.

Putative eORFs of at least 250 codons were predicted using the getorf program (EMBOSS; [95]) and examined for their location either upstream or downstream of the polyprotein coding region. Putative eORFs that were separated from the polyprotein coding region in some elements but were part of it in the others were removed from the analysis. In addition, eORFs present at sequence regions that contained tandem repeats composed of at least three monomers spanning at total of more than 150 bp were excluded because the occurrence of such ORFs was likely due to a lack of stop codons in these low complexity sequence regions. Tandem repeats were predicted using Tandem Repeats Finder [96].

Multiple sequence alignments were calculated using muscle [87] and edited manually in SeaView [97]. In order to decrease the redundancy of the protein domains dataset prior to the phylogenetic analyses, the sequences of the PROT, RT, RH and INT domains of each element were concatenated and subjected to all-to-all blastp comparisons. Sequences that shared at least 80% identity over at least 90% of their length were clustered together. Sequences in each cluster were further compared and clustered using CD-HIT [98] (cd-hit parameters were as follows: -c 0.90 -n 2 -G 1 -g 1 -b 20 -s 0.0 -aL 0.0 -aS 0.0 -S 0) to select a sequence best representing given cluster. Phylogenetic analyses based on maximum likelihood and neighbor-joining algorithms were carried out using PhyML-SMS [99, 100] and BioNJ programs [101], respectively. Phylogenetic trees were visualized and edited in FigTree [102] and Dendroscope [103].

## Additional files

Additional file 1: Information about LTR-retrotransposon sequences included in the study. (XLS 9823 kb)

Additional file 2: A comparison of all protein domains identified in this study with CDD. (PDF 59 kb)

Additional file 3: Sequences of all polyprotein domains in FASTA format. Individual types of domains are distinguished by a prefix which is followed by a name of DNA sequence of the complete element. For example Ty1-RT__REXdb_ID3879 is a protein sequence of RT domain from Ty1/copia element whose entire DNA sequence name is REXdb_ID3879. Information about sources of DNA sequences and classification of complete elements is provided in the Additional file 1. (FASTA 13368 kb)

Additional file 4: Similarity of eORFs to CDD sequences. (PDF 23 kb)

Additional file 5: Summary of features characteristic of individual groups of LTR-retrotransposons. (XLS 17 kb)

Additional file 6: Unrooted neighbor-joining trees inferred from alignments of concatenated alignments of RT-RH-INT (a), and separate alignments of RT (b), RH (c), and INT (d) sequences. Note that chromovirus and non-chromovirus lineages are clearly distinguished in all four trees. Individual clades shown in the Fig. 2 were found on distinct branches yet their mutual positions were partially discordant. Branches that were in conflict with the proposed classification of Ty3/gypsy elements had low bootstrap support values (< 50). (PDF 101 kb)

Additional file 7: Sequence logos of CHD chromodomains. Note that the chromodomain sequences are highly divergent both between and within individual groups of chromoviruses. Three sites corresponding to the aromatic cage motif found in HP1-like chromodomains [50] are marked with triangles if the aromatic amino-acid residues (Y, F, W) are present in most sequences or with crosses if they are mostly absent. The proportion of the aromatic amino-acid residues at the three sites in different groups of chromoviruses is summarized in the table. (PDF 93 kb)

## Abbreviations

aRH: Archeal ribonuclease H
CHD: Chromodomain
CHDCR: Chromodomain of centromeric retrotransposons
eORF: Extra open reading frame
ICTV: The International Committee on Taxonomy of Viruses
INT: Integrase
LTR: Long terminal repeat
ORF: Open reading frame
PBS: Primer binding site
PPT: Polypurine tract
PROT: Protease
RH: Ribonuclease H
RT: Reverse transcriptase
TSD: Target site duplication
