# Supplementary material for: Systematic survey of plant LTR-retrotransposons elucidates phylogenetic relationships of their polyprotein domains and provides a reference for element classification
Source: Mob DNA. 2019 Jan 3;10:1. doi: 10.1186/s13100-018-0144-1 (PMC6317226; doi:10.1186/s13100-018-0144-1)
Supplement: Supplementary file 2 — A comparison of all protein domains identified in this study with CDD. (PDF 59 kb) [file 13100_2018_144_MOESM2_ESM.pdf]

A

Domain type	Number	size range (average) [aa]	CDD domain / superfamily	CDD description	Number of hits to CDD with e-value < 10 / < 1e-10
Ty1-GAG	4968	52-179 (93)	pfam14223 / cl26047	gag-polypeptide of LTR copia-type	4708 / 3487
			pfam03732 / cl04237	Gag or Capsid-like proteins from LTR retrotransposons	1147 / 5
Ty1-PROT	5410	43-93 (71)	cd00303 / cl11403	Pepsin-like aspartate proteases (retropepsins)	121/0
Ty1-INT	5410	136-262 (197)	pfam13976 / cl16514	GAG-pre-integrase domain	5313 / 1188
			pfam00665 / cl21549	Integrase core domain	5409 / 5343
Ty1-RT	5410	183-285 (257)	pfam07727 / cl06662	Reverse transcriptase	5410 / 5410
Ty1-RH	5410	81-200 (127)	cd09272 / cl14782	Ty1/Copia family of RNase HI	5410 / 5405
Ty3-GAG	8294	75-168 (124)	pfam03732 / cl04237	Gag or Capsid-like proteins from LTR retrotransposons	8061 / 5291
Ty3-PROT	8452	56-98 (77)	cd00303 / cl11403	Pepsin-like aspartate proteases (retropepsins)	8408 / 4184
Ty3-RT	8453	107-199 (176)	cd01647 / cl02808	Reverse transcriptase	8452 / 8452
			pfam00078 / cl26764	Reverse transcriptase	8452 / 8274
Ty3-RH	8453	82-381 (152)	cd09274 / cl14782	Ty3/Gypsy family of RNase HI	8450 / 8310
Ty3-INT	8453	135-399 (310)	pfam00665 / cl21549	Integrase core domain	8432 / 6808
Ty3-CHDCR	445	30-131 (61)	-	-	-
Ty3-CHDI/II	3417	59-104 (76)	cd00024, pfam00385, smart00298 / cl17459	Chromatin organization modifier (chromo) domain	3326 / 1369
Ty3-aRH	2941	76-143 (121)	cd09279 / cl14782	RNAse HI family that includes archaeal, some bacterial as well as plant RNase HI	2941 / 2940
			pfam13456 / cl14782	Reverse transcriptase-like domain	2941 / 2926

B

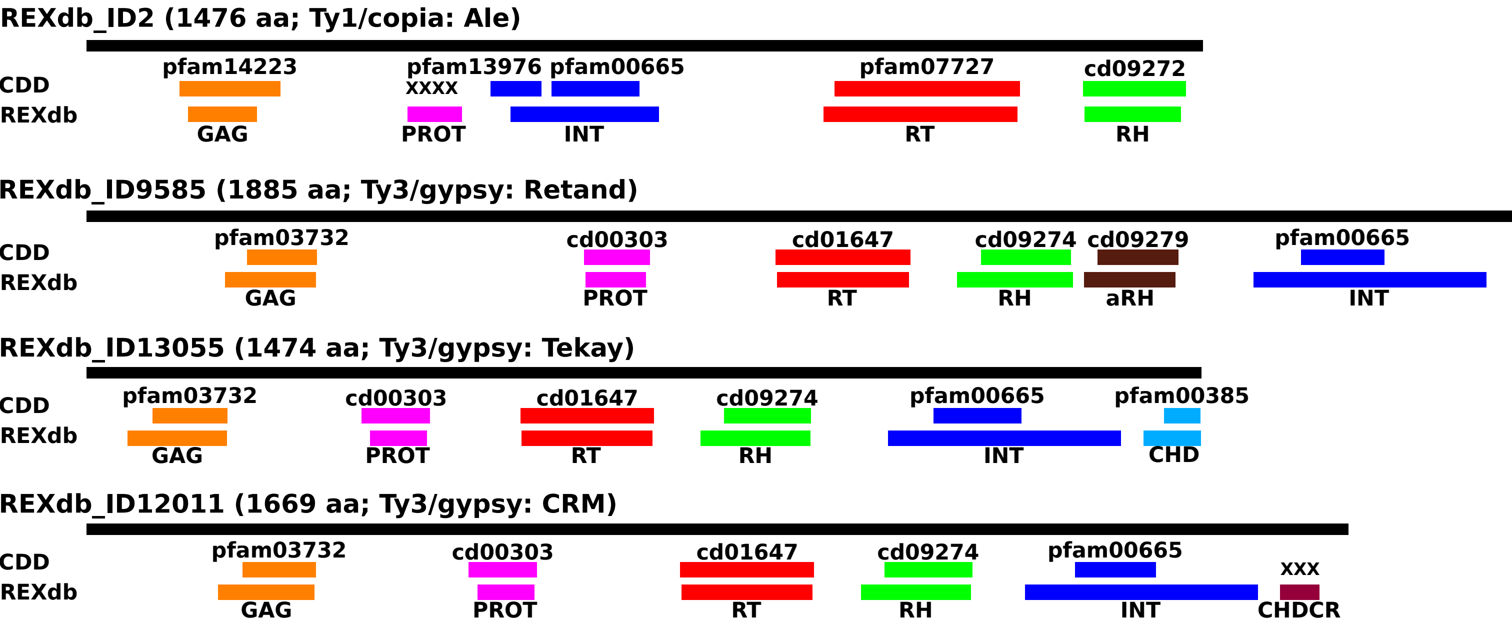

a Results of a CD-search comparison of all protein domains identified in this study with CDD. Most sequences of polyprotein domains, except PROT and CHDCR domains of Ty1/copia and Ty3/gypsy elements, respectively, were detected using CD-search. Failure of CD-search to detect two domains with considerable differences in the number of hits between two different E-value cut-offs suggest that some sequence variants of GAG, PROT, CHD and CHDCR domains are underrepresented in the CDD. **b** Examples of domain detection in putative polyprotein sequences using CD-search compared with positions of individual domains identified in this study. Note that CD-search did not detect PROT and CHDCR domain in the Ty1/copia and CRM chromovirus element, respectively. Also note that the sizes of individual domains differ between CDD and this study. The largest differences were found in the INT domain, which was defined as a region possessing not only the core domain (pfam00665) but also a zinc-finger domain (HHCC) at the N-terminus and a putative DNA-binding domain at the C-terminus which was in Ty3/gypsy elements extended to GPY/F domain.
