# Supplementary material for: Systematic survey of plant LTR-retrotransposons elucidates phylogenetic relationships of their polyprotein domains and provides a reference for element classification
Source: Mob DNA. 2019 Jan 3;10:1. doi: 10.1186/s13100-018-0144-1 (PMC6317226; doi:10.1186/s13100-018-0144-1)
Supplement: Supplementary file 4 — Similarity of eORFs to CDD sequences. (PDF 23 kb) [file 13100_2018_144_MOESM4_ESM.pdf]

Similarity of eORFs to CDD sequences.

CDD domain / superfamily	CDD description	number of elements with hit to CDD	distribution of hits among different lineages of LTR retrotranspons [eORF type: number of hits]
pfam03078 / cl20242	ATHILA ORF-1 family (unknown function)	167	Athila [3'F: 166 hits; 3'R: 1 hit; 5'R: 1 hit]
pfam03641 / cl22881	Possible lysine decarboxylase	95	Athila [3'F]
COG1611/ cl22881	Predicted Rossmann fold nucleotide-binding protein	95	Athila [3'F]
TIGR00730 / cl22881	proteins of unknown function	95	Athila [3'F]
pfam04195 / cl04444	Transposase_28	506	Retand [3'R]
pfam10536 / cl11168	Plant mobile domain	434	Ogre [3'R: 431 hits; 5'F: 3 hits]
pfam04094 / cl25642	Protein of unknown function (DUF390)	164	Retand [3'R]
TIGR02168 / cl25732	chromosome segregation protein SMC, common bacterial type	19	Retand [3'R: 13 hits], Ogre [5'F: 5 hits], Athila [3'F: 1 hit]
pfam04642 / cl27239	Protein of unknown function (DUF601)	16	Retand [3'R]
pfam06721 / cl05990	Protein of unknown function (DUF1204)	15	Retand [3'R]
COG1196 / cl25732	Chromosome segregation ATPase	13	Retand [3'R:9 hits], Ogre [5'F: 4 hits]
pfam07794 / cl25927	Protein of unknown function (DUF1633)	7	Retand [3'R]

The table shows only domains which were detected using CD-search (e-value ≤ 1e-10) in at least five elements.
