# Supplementary figures and images for: Systematic survey of plant LTR-retrotransposons elucidates phylogenetic relationships of their polyprotein domains and provides a reference for element classification
Source: Mob DNA. 2019 Jan 3;10:1. doi: 10.1186/s13100-018-0144-1 (PMC6317226; doi:10.1186/s13100-018-0144-1)

A: RT-RH-INT

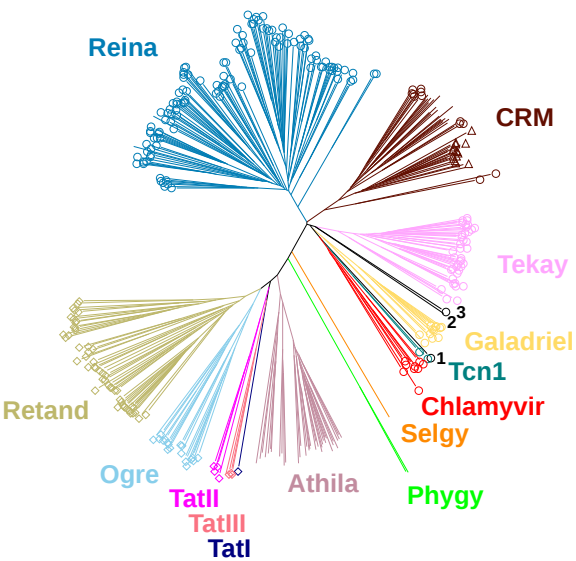

B: RT

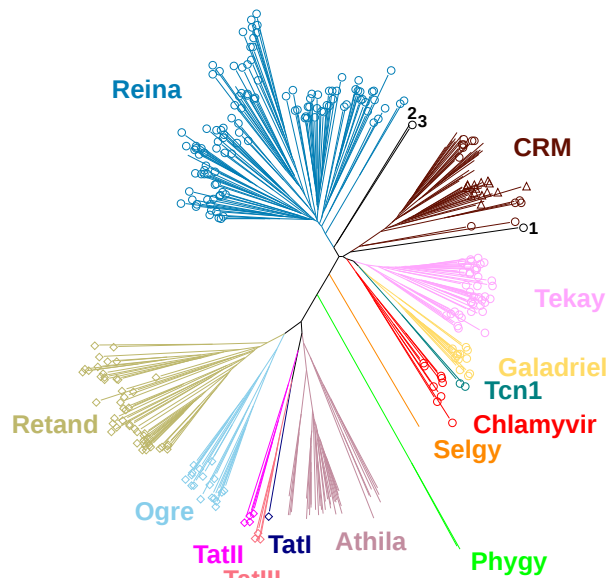

C: RH

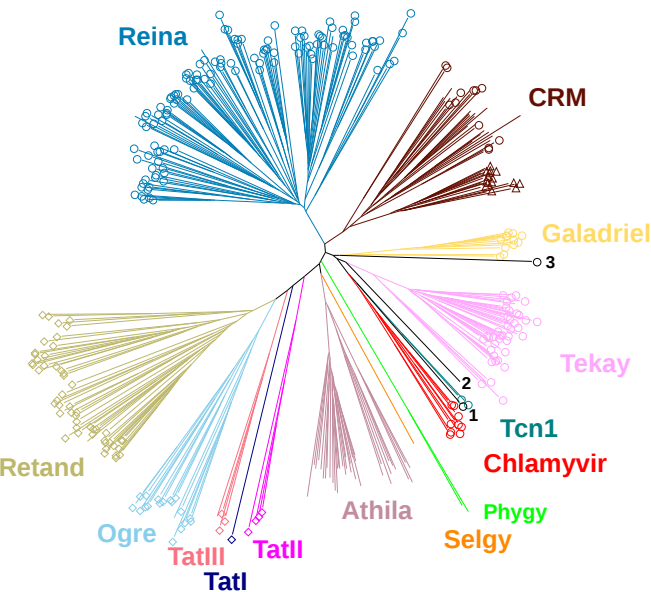

D: INT

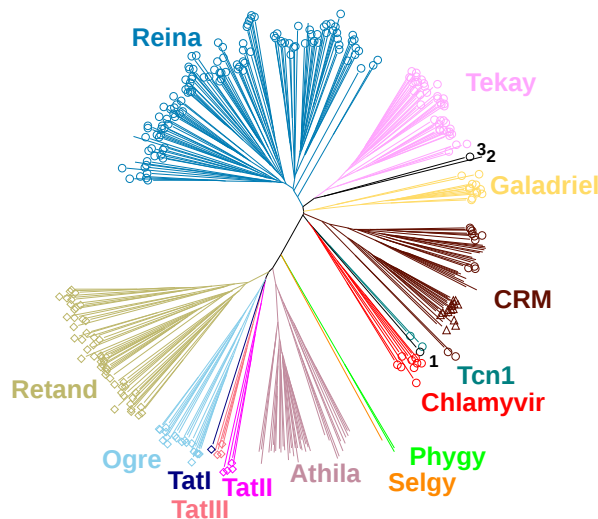

○ CHD    △ CHDCR    ◇ aRH

Supplement: Supplementary file 6 — Unrooted neighbor-joining trees inferred from alignments of concatenated alignments of RT-RH-INT (a), and separate alignments of RT (b), RH (c), and INT (d) sequences. Note that chromovirus and non-chromovirus lineages are clearly distinguished in all four trees. Individual clades shown in the Fig. 2 were found on distinct branches yet their mutual positions were partially discordant. Branches that were in conflict with the proposed classification of Ty3/gypsy elements had low bootstrap support values (< 50). (PDF 101 kb) [file 13100_2018_144_MOESM6_ESM.pdf]

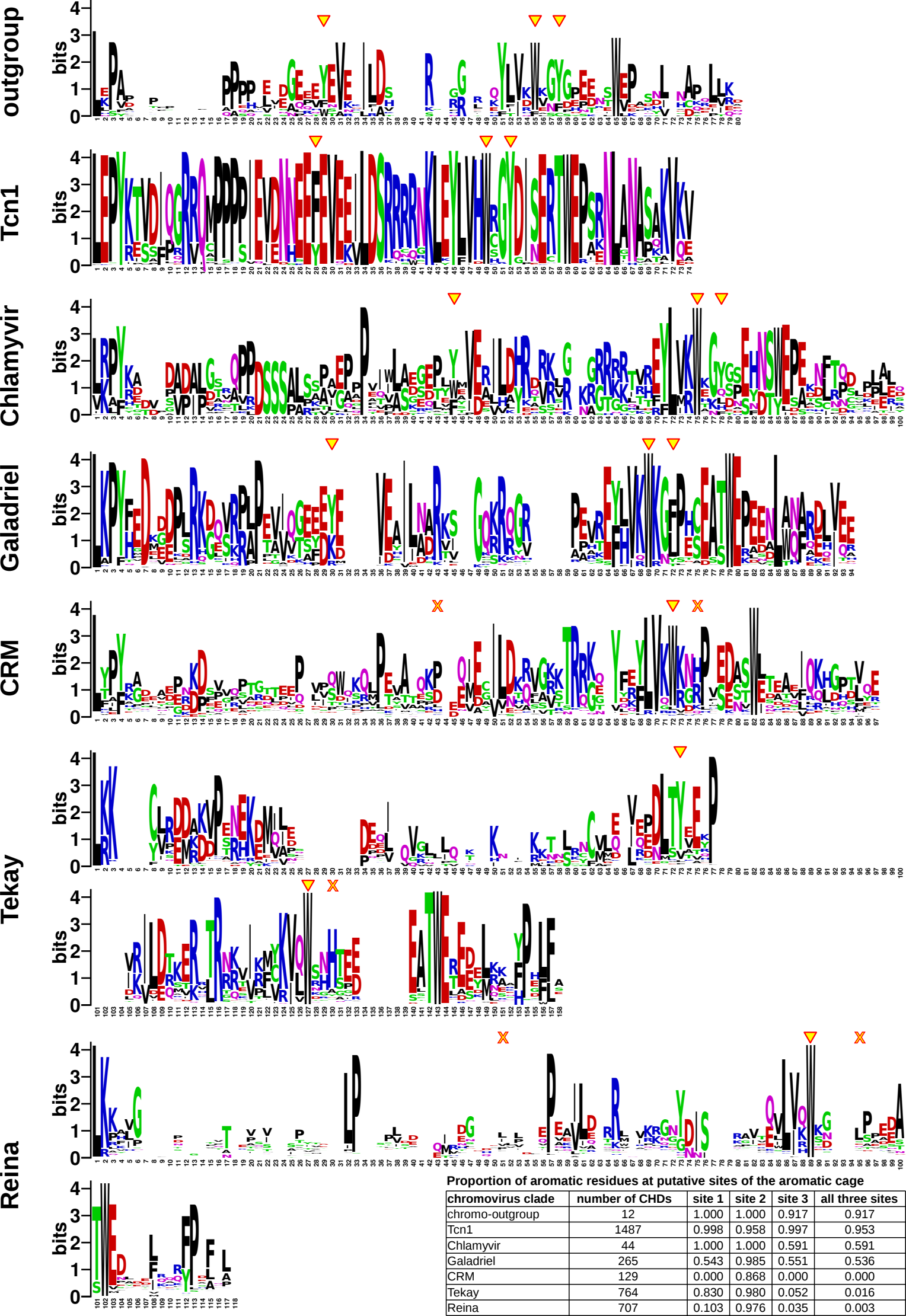

Supplement: Supplementary file 7 — Sequence logos of CHD chromodomains. Note that the chromodomain sequences are highly divergent both between and within individual groups of chromoviruses. Three sites corresponding to the aromatic cage motif found in HP1-like chromodomains [50] are marked with triangles if the aromatic amino-acid residues (Y, F, W) are present in most sequences or with crosses if they are mostly absent. The proportion of the aromatic amino-acid residues at the three sites in different groups of chromoviruses is summarized in the table. (PDF 93 kb) [file 13100_2018_144_MOESM7_ESM.pdf]
